# Size- and Time-Dependent Impacts of Polyvinyl Chloride Microplastics on Turbot (*Scophthalmus maximus* L.): Intestinal Tolerance, Hepatic Injury, and Intestinal Microbiota Dysbiosis

**DOI:** 10.3390/toxics14040321

**Published:** 2026-04-12

**Authors:** Xiaoyang Yao, Jinzhu Yang, Kangsen Mai, Yanjiao Zhang

**Affiliations:** 1 The Key Laboratory of Aquaculture Nutrition and Feed (Ministry of Agriculture and Rural Affairs), The Key Laboratory of Mariculture (Ministry of Education), Ocean University of China, 5 Yushan Road, Qingdao 266003, China; yaoxiaoyang@stu.ouc.edu.cn (X.Y.); yangjinzhu@stu.ouc.edu.cn (J.Y.); kmai@ouc.edu.cn (K.M.); 2 Qingdao National Laboratory for Marine Science and Technology, 1 Wenhai Road, Qingdao 266237, China

**Keywords:** microplastics, particle size, exposure duration, intestine, liver, turbot

## Abstract

The present study aimed to investigate the effects of polyvinyl chloride microplastics with different sizes on the growth, intestinal and hepatic health of turbot (*Scophthalmus maximus* L.) at 3 and 9 weeks of exposure. Three diets were formulated: a control diet with no microplastics, a diet containing 2% micrometer-sized plastics (MPs), and a diet containing 2% nanoplastics (NPs), with four replicates (40 fish/tank, 12 tanks total). The results showed that MPs and NPs had no significant effects on the growth performance of turbot. Analyses of intestinal histology and gene expression (intestinal barrier-related and antioxidant-related genes) indicated that the turbot intestine exhibited a certain degree of tolerance and adaptability to MPs and NPs exposure. Observations of liver histology and analyses of gene expression (inflammatory cytokines, apoptosis-related, and antioxidant-related genes) revealed that the liver damage induced by microplastics in turbot exhibited obvious size-dependent and time-cumulative effects, with NPs exerting a stronger impact. Compared with MPs, long-term exposure to NPs can induce obvious intestinal microbiota dysbiosis in turbot. In summary, particle size and exposure duration are important factors regulating the impacts of PVC microplastics on the intestinal and hepatic health of turbot.

## 1. Introduction

Microplastics generally refer to plastic particles with a diameter of less than 5 mm [1], which can be classified by particle size into micrometer-sized plastics (MPs) and nanoplastics (NPs) [2]. Most microplastics are difficult to completely degrade [3], and due to their small particle size, they are highly susceptible to ingestion by aquatic organisms [4]. The chemical additives contained within microplastics themselves [5], as well as heavy metals and organic pollutants adsorbed on their surfaces, can be transferred and accumulated along the food chain [6]. This not only disrupts the balance of aquatic ecosystems but also poses long-term health risks to various organisms, including humans [7]. With the continuous increase in aquaculture production [8] and the extensive use of plastic products [9], the risks of microplastics pollution in aquaculture environments have gradually risen. To date, several studies have detected microplastics in aquaculture environments [10,11,12].

The intestines and liver are core target organs of microplastic exposure, and their functional homeostasis is crucial for maintaining fish health [13]. The intestine acts as both the primary site for the digestion and absorption of nutrients and a key immune barrier against exogenous pollutants. After entering the fish body via ingestion, microplastics tend to accumulate in the intestine [14], thereby impairing intestinal health [15,16]. As an important organ responsible for the synthesis and metabolism of nutrients, as well as the decomposition and clearance of wastes and toxicants, the liver is prone to hepatocellular damage due to the accumulation of microplastics and their associated harmful substances [17,18]. Furthermore, the intestine harbors a complex and dynamically changing microbial community that plays a critical role in maintaining intestinal homeostasis [19]. Microplastic exposure is likely to induce intestinal dysbiosis [20,21], and the intestinal microbiota can serve as a significant biomarker for assessing microplastic toxicity.

The particle size of microplastics is a key factor influencing their toxic effects on fish [22]. Theoretically, microplastics with smaller particle sizes are more likely to penetrate the intestinal barrier and translocate to other tissues [14,23], and even disrupt the structure of biomolecules [24]. Some studies have shown that smaller-sized microplastics exhibit greater toxicity, primarily manifested in inducing tissue damage [25] and inflammation [26], as well as triggering oxidative stress [27]. Meanwhile, the toxic effects of microplastics on aquatic animals are also affected by exposure duration. Studies have shown that the degree of damage caused by microplastics to aquatic animals increases with the extension of exposure duration [28,29]. However, most current studies are still limited to the exploration of single factors, and few have conducted systematic comparative analyses by combining particle size and exposure time.

Polyvinyl chloride (PVC) microplastics are a significant component of microplastics in natural environments. Although their abundance in aquaculture environments and aquatic organisms is generally lower than that of common polymers such as polyethylene and polypropylene—for example, 6.2% and 11.4% in water and sediments of the Poyang Lake, China [30], 7.16% and 3.58% in water and fish from aquaculture ponds in Bangladesh [31], and 5.52% in *Litopenaeus vannamei* from the Jiulong River, China [32]—PVC microplastics exhibit a significantly higher hazard risk index than other types of microplastics [32]. PVC microplastics can impair the health of farmed fish through multiple pathways, including inducing intestinal dysbiosis in carp (*Cyprinus carpio*) [20], exacerbating stress levels and oxidative damage in Nile tilapia (*Oreochromis niloticus*) [33], and causing hepatic dysfunction in *Sebastes schlegelii* [34]. Therefore, PVC microplastics represent high-risk pollutants that warrant focused attention in aquaculture. As an important farmed fish worldwide, turbot (*Scophthalmus maximus* L.) has high economic value, but research on the health impacts of microplastics on it remains relatively scarce. To address this gap, the present study incorporated PVC microplastics of two different particle sizes into the feed. By analyzing the growth performance, histomorphological characteristics and gene expression of the intestine and liver, as well as the composition and structure of the intestinal microbiota of turbot at two rearing time points (3rd week and 9th week), we aimed to investigate the particle size- and time-dependent effects of PVC microplastics on turbot health. This work is expected to provide a theoretical basis for evaluating the toxic effects of microplastics on aquatic animals.

## 2. Materials and Methods

### 2.1. Experimental Diets

Three diets were formulated: a diet without microplastic addition (diet C), a diet supplemented with 2% by mass pure PVC MPs (average diameter: approximately 63 μm; diet M), and a diet supplemented with 2% by mass pure PVC NPs (average diameter: approximately 582 nm; diet N). A 2% microplastic addition in feed is a commonly used dose in aquatic microplastic toxicology research, as it can induce toxic effects within a limited experimental period [35,36,37], thereby facilitating the clarification of the harmful impacts of microplastics on the intestinal and hepatic health of turbot. Furthermore, considering that the culture cycle of turbot typically ranges from 10 to 15 months, short-term exposure to high doses can simulate the cumulative effects of long-term low-dose exposure. The microplastics were purchased from a factory producing raw materials for PVC pipes and stored in sealed containers at room temperature until use. The morphological characteristics of the microplastics were determined using a Phenom ProX G6 Desktop SEM (Thermo Fisher Scientific, Waltham, MA, USA) (Figure S1A,B), and the particle diameters were measured using Image-Pro Plus^®^ (version 6.0.0.260, Media Cybernetics, Silver Spring, MD, USA) (Figure S1C,D).

The general process of diet formulation was as follows: The microplastics were thoroughly mixed with water and then added to the comminuted commercial turbot feed produced by Qingdao Great-seven Nutr-tech Co., Ltd. (Qingdao, Shandong, China). After thorough mixing, the mixture was made into feed pellets with a diameter of approximately 3 mm through a granulator. These pellets were then dried in an oven at 55 °C to a constant weight and stored at −20 °C.

### 2.2. Feeding Trial

Juvenile turbot were purchased from a commercial farm in Haiyang (Yantai, Shandong, China). The feeding trial was conducted at Haiyang Yellow Sea Fisheries Co., Ltd. (Yantai, Shandong, China). Prior to the start of the experiment, the juvenile fish were fed with commercial feed for two weeks to acclimate to the experimental environment. After 24 h of fasting, 480 healthy juvenile fish with similar specifications were selected, weighed (initial body weight: 5.90 ± 0.005 g), and randomly allocated to 12 tanks with 40 fish per tank. The three experimental diets were randomly assigned to 12 tanks, with each diet allocated to 4 replicate tanks. The group fed with diet C served as the control group, the group fed with diet M as the MPs group, and the group fed with diet N as the NPs group. During the feeding trial, the fish were fed twice a day (8:00 and 18:00) to apparent satiation. Feces were removed half an hour after each feeding, and two-thirds of the volume of seawater was replaced twice a day. The feeding experiment lasted for nine weeks. During the culture period, the water temperature was 15–21 °C, the salinity was 31–34, the pH was 7.9–8.2, and the dissolved oxygen was greater than 7 mg/L.

### 2.3. Sample Collection

Samples were collected at two culture time points (3rd week and 9th week). Based on the type of feed administered and sampling time, the samples were divided into 6 groups, labeled as C3, M3, N3, C9, M9, and N9, respectively. Prior to sample collection, the juvenile fish were fasted for 24 h. The number of all surviving fish was recorded, and their body weights were measured. In the present study, each individual fish was regarded as an independent biological replicate. Two fish were randomly selected from each tank (8 fish per group in total), the posterior intestine samples were dissected, collected into 1.8 mL sterile enzyme-free tubes (Cat. No.: 377267, Nunc™, Rochester, NY, USA), immediately placed in liquid nitrogen, and stored at −80 °C for real-time fluorescence quantitative PCR (qPCR) analysis. Another two fish were randomly selected from each tank (8 fish per group in total). After wiping the body surface with 70% alcohol, the whole intestine (with pyloric caeca and cecum removed) and liver were dissected using sterile dissecting tools near an alcohol lamp, then transferred into 1.8 mL sterile enzyme-free tubes (Cat. No.: 377267, Nunc™, Rochester, NY, USA). These samples were immediately placed in liquid nitrogen and stored at −80 °C, with the whole intestine used for intestinal microbiota DNA extraction and the liver for qPCR analysis. Additionally, two fish were randomly selected from each tank (8 fish per group in total). The liver and posterior intestine were dissected, fixed in Bouin’s fixative for 24 h, and then transferred to 70% alcohol for storage, which were used for the preparation of hematoxylin–eosin (HE)-stained tissue sections. All experimental fish were anesthetized with eugenol (1:10,000, purity 99%, Shanghai Reagent, Shanghai, China) prior to sampling.

### 2.4. Growth Performance Equations

Table growth performance is calculated by the following equations:

(1) $$\mathrm{WGR}\;(\%)=(W_{f}-W_{i})/W_{i}\times 100$$

(2) $$SGR\;(\%/day)=(\ln W_{f}-\ln W_{i})/d\times 100$$

(3) $$FE=(W_{f}-W_{i})/F$$

(4) $$FI\;(\%/day)=(2\times F)/[\left(W_{f}+W_{i}\right)\times days]\times 100$$

(5) $$SR\;(\%)=N_{f}/N_{i}\times 100$$

wherein, WGR refers to weight gain rate, SGR to specific growth rate, FE to feed efficiency, FI to feed intake rate, and SR to survival rate. W_f_ refers to final body weight, W_i_ initial body weight, F to feed intake, N_f_ to final number of turbot, and N_i_ to initial number of turbot.

### 2.5. Histological Analysis

The posterior intestine and liver tissues were dehydrated and paraffin-embedded, then sections with a thickness of 5 μm were cut using a paraffin microtome (Leica RM2235, Wetzlar, Hesse, Germany). These slices were then stained with hematoxylin and eosin (HE) (BC-DL-001, Sbjbio, Nanjing, Jiangsu, China). The tissue sections were observed under an optical microscope (DP72, Olympus, Hachioji, Tokyo, Japan), and image acquisition was carried out using the camera (E600, Nikon, Shinagawa, Tokyo, Japan) equipped with it and CellSens Standard Software (version 2.2, Olympus, Hachioji, Tokyo, Japan). The intestinal villus height, lamina propria thickness, muscular thickness, and perimeter ratio (PR) were measured using Image-Pro Plus (version 6.0.0.260, Media Cybernetics, Silver Spring, MD, USA). The PR was defined as the ratio of the internal perimeter (IP) of the intestinal lumen to the external perimeter (EP) of the intestine (PR = IP/EP) [38].

### 2.6. RNA Extraction and qPCR

Total RNA from the intestines and livers was extracted using Trizol reagent (Cat. No.: TR201-100, Genstone Biotech, Beijing, China). Subsequently, intestinal RNA was purified with the SteadyPure RNA Extraction Kit (Cat. No.: AG21024, Accurate Biology, Changsha, Hunan, China), and liver RNA was purified using the Tirzol Total RNA Rapid Extraction Kit (chloroform-free) (Cat. No.: TR205-200, Genstone Biotech, Beijing, China). The integrity of the extracted RNA was determined by electrophoresis on a 1.2% (*w*/*v*) agarose gel. The concentration and quality of the RNA were evaluated using a NanoDrop™ 2000 spectrophotometer (Thermo Scientific™, Waltham, MA, USA). Then, RNA was reverse transcribed into cDNA using the 5× All-In-One RT MasterMix (with AccuRT Genomic DNA Removal Kit) Kit (Cat. No.: G492, Applied Biological Materials Inc., Richmond, BC, Canada) and a PCR instrument (Biometra Tone 96G, Analytik Jena AG, Jena, Thuringia, Germany).

All specific gene primers were designed in NCBI (Table S1) and synthesized by Sangon Biotech (Shanghai) Co., Ltd. (Shanghai, China). Prior to the formal qPCR experiment, reference genes were screened. Specifically, the expression levels of three commonly used reference genes, *β-actin*, glyceraldehyde-3-phosphate dehydrogenase (*GAPDH*), and ribosomal protein L17 (*RPL17*), in all intestinal and liver samples were detected by qPCR. Subsequently, RefFinder (http://blooge.cn/RefFinder) (accessed on 18 August 2025) was employed to evaluate the stability of the three reference genes (Table S2) [39,40]. Eventually, *RPL17* was selected as the reference gene for the formal experiment. The total volume of all qPCR systems was 20 μL, consisting of 4 μL of cDNA product, 0.25 μL of 20 μM forward primer, 0.25 μL of 20 μM reverse primer, 10 μL of BlasTaq™ 2× qPCR MM (Cat. No.: G891, Applied Biological Materials Inc., Richmond, BC, Canada), and 5.5 μL of DEPC-treated water (Sangon biotech, Shanghai, China). The reaction program for qPCR was as follows: 95 °C for 3 min and then 40 cycles of 95 °C for 10 s and 60 °C for 30 s. The specification of the amplified products was ensured by melting curve analysis. A total of three quantitative thermal cyclers were used in the qPCR experiment: the CFX96 Touch™ Real-Time PCR Detection System (Bio-Rad, Richmond, CA, USA), the Pangaea Rapid Fluorescent Quantitative PCR System (Aperbio, Suzhou, Jiangsu, China), and the QuantStudio™ 5 Real-Time PCR System (Applied Biosystems, Foster City, CA, USA). The relative expression levels of genes were calculated using the 2^−ΔΔCq^ method [41].

### 2.7. DNA Extraction and Sequencing of Intestinal Microbiota

Genomic DNA was extracted from the intestinal mucosa using the DNeasy^®^ PowerSoil^®^ Pro Kit (Cat. No.: 47,014, Qiagen, Hilden, North Rhine-Westphalia, Germany) in a laminar flow cabinet, adjacent to the flame of an alcohol lamp. Meanwhile, ZymoBIOMICS^®^ Microbial Community Standard (D6300, Zymo Research Corp, Irvine, CA, USA) was used as a positive control (PC), and a no-sample negative control (NC) was set up [42]. Subsequently, the samples were sent to Novogene Genomics Technology Co., Ltd. (Beijing, China) for sequencing. The brief operational procedures were as follows: The V4 region of the 16S rRNA gene was amplified using the 515F/806R primers. After the PCR products passed the quality inspection, a library was constructed. Once the library passed the quality inspection, sequencing was performed on the Illumina NovaSeq platform. On the NovoMagic cloud platform, the original sequencing data underwent quality control (removing low-quality sequences and splitting the mixed data according to the Barcode) and denoising (removing primer and chimera sequences using the DADA2 algorithm based on the QIIME2 platform) [43]. Subsequently, an amplicon sequence variant (ASVs) table and a feature sequence file were generated.

In R (v.4.3.2), ASVs with a single frequency were filtered using the R package *dplyr* (version 1.1.3) [44]. Species annotation was carried out using the R package *dada2* (version 1.26.0) [45] based on the Silva 138.2 database [46]. Subsequently, ASVs and characteristic sequences annotated as Archaea, Eukaryota, Unassigned (kingdom and phylum levels), Chloroplast, and Mitochondria were removed. In cases where annotation to a specific taxonomic unit was not possible, the lowest level taxonomic information that could be annotated was filled in, preceded by the prefix letter of the corresponding taxonomic unit. Subsequently, contaminating sequences were removed using the R package *decontam* (version 1.18.0), which operates on the principle that contaminant sequences are frequently present in negative controls and their abundance is generally inversely proportional to the concentration of bacterial DNA in the samples [47]. Quantitative detection of bacterial DNA in all samples except the positive control (PC) and negative control (NC) was carried out by qPCR [48,49]. The experiment was conducted in a laminar flow cabinet near the flame of an alcohol lamp. The total volume of the qPCR system was 20 μL, consisting of 2 μL of DNA sample, 0.6 μL of 10 μM forward primer (5′-CCATGAAGTCGGAATCGCTAG-3′), 0.6 μL of 10 μM reverse primer (5′-GCTTGACGGGCGGTGT-3′), 1 μL of SYBR Green I (20×, Cat. No.: SL2190, Coolaber, Beijing, China), 10 μL of TaKaRa Taq™ HS Low DNA (2×, Cat. No.: R090A, Takara Biotech, Beijing, China), and 5.8 μL of DEPC-treated water (Sangon biotech, Shanghai, China). A no-sample negative control (NTC) was also set up to ensure that there was no exogenous DNA contamination during the operation. The qPCR reaction program was 40 cycles of 94 °C for 5 s and 60 °C for 20 s. The specificity of the amplified products was ensured by melting curve analysis. Since the concentration of bacterial DNA is positively correlated with 10^−Cq^, this value was used to indicate the concentration of bacterial DNA in the samples [50]. A rooted phylogenetic tree was constructed using Wekemo Bioincloud (https://www.bioincloud.tech) (accessed on 24 October 2025) [51].

All data visualizations were carried out in R. The R packages *BiodiversityR* (version 2.15-4) [52], *doBy* (version 4.6.27) [53], *ggplot2* (version 3.5.2) [54], and *vegan* (version 2.6-4) [55] were used to plot species accumulation boxplots, rarefaction curves, and rank abundance curves. The R packages *ggalluvial* (version 0.12.5) [56], *ggplot2*, and *tidyverse* (version 2.0.0) [57] were employed to draw stacked bar plots of the relative abundances of the top 10 species at the phylum level and the top 30 species at the genus level. The R packages *ape* (version 5.8-1) [58], *dplyr*, *ggplot2*, *ggtree* (version 3.6.2) [59], *phyloseq* (version 1.42.0) [60], and *treeio* (version 1.22.0) [61] were used to compute unweighted UniFrac distance matrices and construct an unweighted pair group method with arithmetic mean (UPGMA) cluster tree. Principal coordinates analysis (PCoA) was performed and plotted using the R packages *ape*, *dplyr*, *ggplot2*, *GUniFrac* (version 1.8) [62], *phyloseq*, *plyr* (version 1.8.9) [63], and *vegan*. Simultaneously, Adonis tests and multi response permutation procedure (MRPP) were conducted. A genus level microbial co-occurrence network was constructed using the R packages *ggClusterNet* (version 2.00) [64] and *ggplot2*. Features with an average relative abundance within each group less than 0.01% were filtered out, and only edges with a correlation coefficient |r| > 0.6 and *p* < 0.05 were retained. Subsequently, the robustness and invulnerability of the microbial co-occurrence network were evaluated using the *Robustness.Random.removal* and *natural.con.microp* functions, respectively.

### 2.8. Statistical Analysis

Growth performance, intestinal tissue morphological parameters, and qPCR results were statistically analyzed using SPSS 22.0 (IBM SPSS corporation, Chicago, IL, USA). When the data met the normality (Shapiro–Wilk test, *p* > 0.05) and homogeneity of variance (Levene test, *p* > 0.05), for horizontal comparisons (comparisons among different treatments at the same time), one-way analysis of variance (ANOVA) was employed, and Tukey’s test was used to compare the differences among different groups. A significant difference was considered when *p* < 0.05. For longitudinal comparisons (comparisons at different times within the same treatment), an independent samples t-test was used, and a significant difference was considered when *p* < 0.05. When the data met the normality but not the homogeneity of variance, Welch’s ANOVA was used for horizontal comparisons, and Games–Howell test was applied to compare the differences among different groups. A significant difference was considered when *p* < 0.05. For longitudinal comparisons, Welch’s test was used, and a significant difference was considered when *p* < 0.05. When the data did not meet the normality, non-parametric tests were carried out. For horizontal comparisons, the Kruskal–Wallis test was used, and a significant difference was considered when *p* < 0.05. For longitudinal comparisons, the Mann–Whitney U-test was used, and a significant difference was considered when *p* < 0.05. Finally, the data were presented as mean ± standard error (SE).

The R package *Maaslin2* (version 1.12.0) [65] was used to screen for differential features at the genus level of intestinal microbiota. Features with an occurrence frequency of less than 50% within each group, an average relative abundance of less than 0.1%, and those not annotated to the genus level were filtered out. A significant difference was considered when *q* < 0.05 (*p*–adjust).

## 3. Results

### 3.1. Growth Performance

No significant differences were observed in WGR, SGR (Kruskal–Wallis test: both χ^2^ = 1.423; both df = 2; both *p* = 0.491), FE, FI (one-way ANOVA: F = 0.153 and 1.461; both dfnum = 2; both dfden = 9; *p* = 0.860 and 0.282), and SR (Kruskal–Wallis test: χ^2^ = 0.688; df = 2; *p* = 0.709) of turbot among the C3, M3, and N3 groups. Similarly, there were no significant differences in the above indicators among the C9, M9, and N9 groups (one-way ANOVA: F = 0.993 and 0.942; both dfnum = 2; both dfden = 9; *p* = 0.408 and 0.425) (Kruskal–Wallis test: χ^2^ = 0.476, 0.474 and 0.629; all df = 2; *p* = 0.788, 0.789 and 0.730) (Table 1).

### 3.2. Intestinal Histology

**Figure 1:**
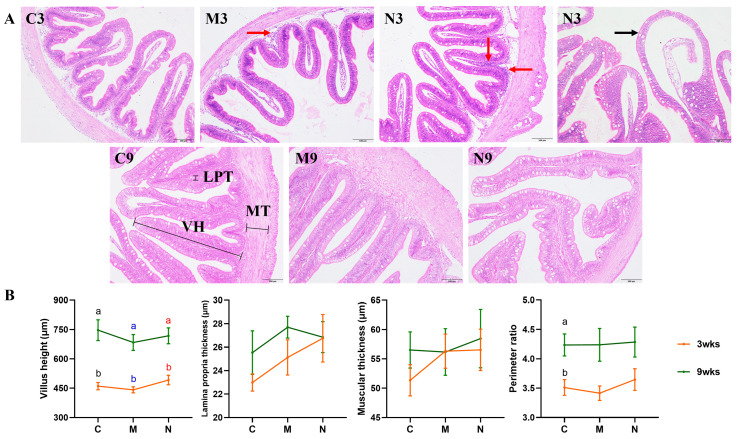
Effects of MPs and NPs on intestinal histology of turbot. Intestinal tissue sections of turbot (**A**). C3, M3 and N3: control, MPs, and NPs groups in the 3rd week; C9, M9 and N9: control, MPs, and NPs groups in the 9th week. Scale bar = 100 µm. Black arrow indicates villus broadening, and red arrows indicate inflammatory cell infiltration. Staining: H&E. Intestinal villus height (VH), lamina propria thickness (LPT), muscular thickness (MT), and perimeter ratio (**B**). C, M and N: control, MPs, and NPs groups. Results are shown as means ± SE, *n* = 8. The value for each fish is expressed as the mean of 10 measurements. ^a,b^ Values with different superscript letters (marked in the same color) indicate significant differences over time within the same treatment (*p* < 0.05).

As shown in Figure 1A, most intestinal villi of turbot in all groups exhibited normal morphology and intact structure. However, obvious inflammatory cell infiltration was observed in both the M3 and N3 groups, with the N3 group being more severe than the M3 group. Additionally, obvious villus broadening was detected in the N3 group. As shown in Figure 1B, there were no significant differences in intestinal villus height, muscular thickness, lamina propria thickness, and perimeter ratio among the C3, M3, and N3 groups (one-way ANOVA: F = 1.431, 0.810, 1.366 and 0.532; all dfnum = 2; all dfden = 21; *p* = 0.261, 0.458, 0.277 and 0.595). Similarly, no significant differences in the above indicators were found among the C9, M9, and N9 groups (one-way ANOVA: F = 0.423, 0.080 and 0.519; all dfnum = 2; all dfden = 21; *p* = 0.661, 0.924 and 0.603) (Kruskal–Wallis test: χ^2^ = 0.035; df = 2; *p* = 0.983).

### 3.3. Intestinal Gene Expression

**Figure 2:**
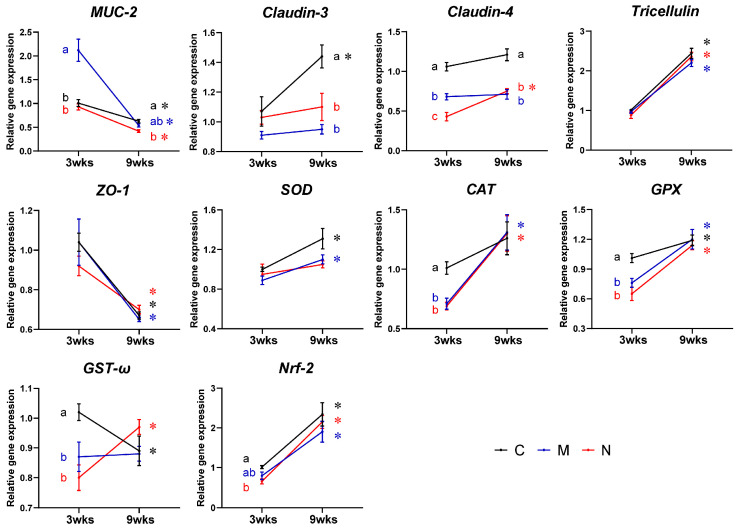
Effects of MPs and NPs on intestinal gene expression in turbot. Results are shown as means ± SE, *n* = 8. C, M and N: control, MPs, and NPs groups. ^a,b,c^ Values with different superscript letters indicate significant differences among treatments at the same time (*p* < 0.05). * Values marked with asterisks indicate significant differences over time within the same treatment (*p* < 0.05).

The results of intestinal gene expression in turbot are shown in Figure 2.

#### 3.3.1. Intestinal Barrier-Related Genes

The expression of mucin-2 (*MUC-2*) in the M3 group was significantly upregulated compared with the C3 and N3 groups (*p* = 0.004 and 0.003), with no significant difference between the C3 and N3 groups (*p* = 0.640) (Welch’s ANOVA: F = 11.553; dfnum = 2; dfden = 12.713; *p* = 0.001). The expression of *Claudin-4* in the M3 and N3 groups was significantly downregulated compared with the C3 group (both *p* < 0.001) (one-way ANOVA: F = 43.540; dfnum = 2; dfden = 21; *p* < 0.001). There were no significant differences in the expressions of *Claudin-3*, *Tricellulin*, and zonula occluden-1 (*ZO-1*) (one-way or Welch’s ANOVA: F = 3.485, 1.130 and 0.817; all dfnum = 2; dfden = 11.622, 12.216 and 21; *p* = 0.065, 0.355 and 0.455) among the C3, M3, and N3 groups. Compared with the C9 group, the expression of *MUC-2* showed no significant difference in the M9 group (*p* = 0.147) but was significantly downregulated to a small extent in the N9 group (*p* = 0.001) (one-way ANOVA: F = 9.546; dfnum = 2; dfden = 21; *p* = 0.001). The expressions of *Claudin-3* (*p* = 0.001 and 0.038) and *Claudin-4* (both *p* < 0.001) in the M9 and N9 groups were significantly downregulated (one-way or Welch’s ANOVA: F = 15.963 and 22.839; both dfnum = 2; dfden = 11.450 and 21; both *p* < 0.001), while there were no significant differences in the expressions of *Tricellulin* and *ZO-1* (one-way ANOVA: F =1.268 and 2.416; both dfnum = 2; both dfden = 21; *p* = 0.302 and 0.114).

#### 3.3.2. Antioxidant-Related Genes

Compared with the C3 group, the expressions of catalase (*CAT*) (both *p* < 0.001), glutathione peroxidase (*GPX*) (*p* = 0.009 and <0.001), and omega class glutathione S-transferase (*GST-ω*) (*p* = 0.043 and 0.003) in the M3 and N3 groups were significantly downregulated (one-way ANOVA: F = 17.226, 11.800 and 7.399; all dfnum = 2; all dfden = 21; *p* < 0.001, <0.001 and =0.004), while the expression of superoxide dismutase (*SOD*) showed no significant difference (one-way ANOVA: F = 2.580; dfnum = 2; dfden = 11.569; *p* = 0.118). For the expression of nuclear factor erythroid 2-related factor 2 (*Nrf-2*), there was no significant difference in the M3 group (*p* = 0.091), but a significant downregulation was observed in the N3 group (*p* = 0.004) (one-way ANOVA: F = 6.885; dfnum = 2; dfden = 21; *p* = 0.005). However, no significant differences in the expressions of the above five genes were found among the C9, M9, and N9 groups (one-way or Welch’s ANOVA: F = 0.032, 0.273, 1.856, 2.879 and 0.799; all dfnum = 2; dfden = 21, 13.156, 21, 12.697 and 21; *p* = 0.969, 0.766, 0.181, 0.093 and 0.463).

### 3.4. Hepatic Histology

**Figure 3:**
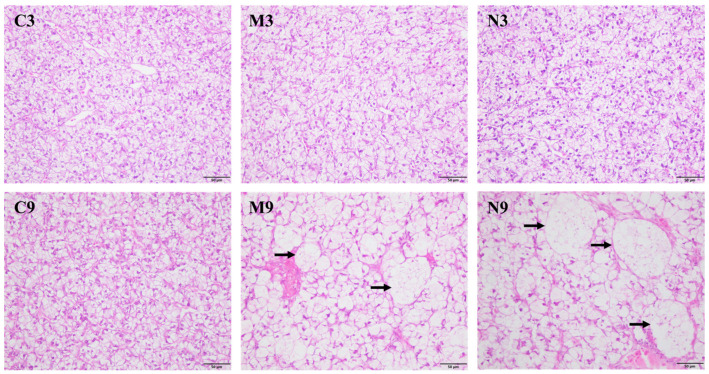
Effects of MPs and NPs on histology of turbot: hepatic tissue sections. C3, M3 and N3: control, MPs, and NPs groups in the 3rd week; C9, M9 and N9: control, MPs, and NPs groups in the 9th week. Scale bar = 50 µm. Black arrows indicate vacuolar structure. Staining: H&E.

As shown in Figure 3, the liver tissues of turbot in the C3, M3, and N3 groups, and the C9 group exhibited intact structures. Hepatocytes were polygonal with clear boundaries and regular arrangement. Cell nuclei were round or oval with distinct nucleoli, and no obvious degeneration, necrosis, or inflammatory cell infiltration was observed. However, in the M9 and N9 groups, hepatocytes were significantly swollen and rounded, with blurred boundaries and disordered arrangement. Nuclear deformation was noted, along with the presence of vacuolar structures, and the condition was more severe in the N9 group than in the M9 group.

### 3.5. Hepatic Gene Expression

**Figure 4:**
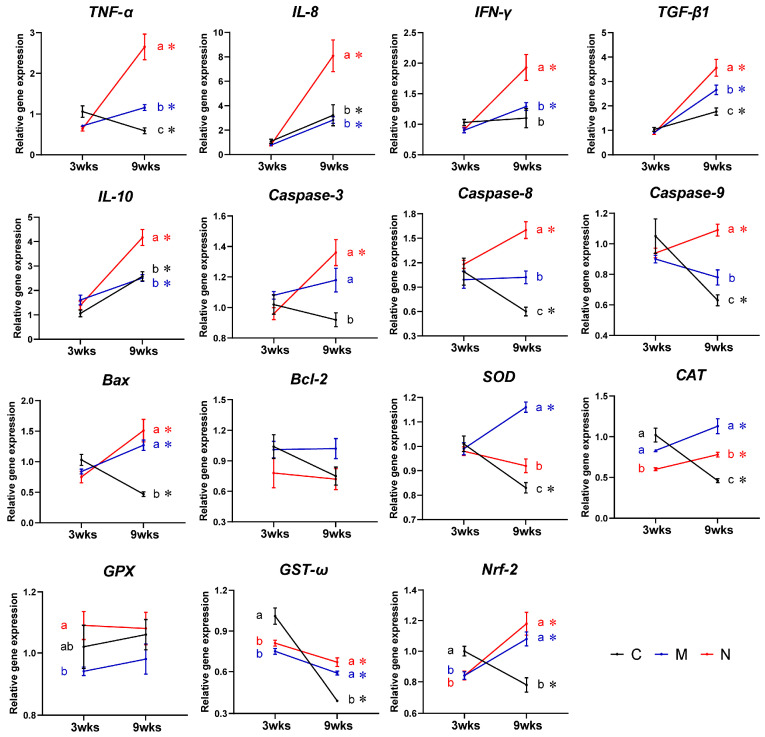
Effects of MPs and NPs on hepatic gene expression in turbot. Results are shown as means ± SE, *n* = 8. C, M and N: control, MPs, and NPs groups. ^a,b,c^ Values with different superscript letters indicate significant differences among treatments at the same time (*p* < 0.05). * Values marked with asterisks indicate significant differences over time within the same treatment (*p* < 0.05).

The results of intestinal gene expression in turbot are shown in Figure 4.

#### 3.5.1. Inflammatory Cytokine Genes

In the horizontal comparison, there were no significant differences in the expressions of tumor necrosis factor alpha (*TNF-α*), interleukin 8 (*IL-8*), interferon gamma (*IFN-γ*), transforming growth factor-beta 1 (*TGF-β1*), and interleukin 10 (*IL-10*) among the C3, M3, and N3 groups (one-way or Welch’s ANOVA: F = 3.590, 1.392, 2.578, 1.735 and 2.512; all dfnum = 2; dfden = 10.049, 13.048, 21, 21 and 21; *p* = 0.067, 0.283, 0.100, 0.201 and 0.105). Compared with the C9 group, the expressions of *TNF-α* and *TGF-β1* in the M9 group were significantly upregulated (*p* < 0.001 and =0.043). However, the expressions of the above five genes in the N9 group were significantly upregulated compared with the C9 groups (*p* = 0.001, 0.021, 0.020, <0.001 and <0.001). The expressions of all five genes in the N9 group were significantly higher than those in the M9 group (*p* = 0.005, 0.011, 0.048, 0.043 and <0.001) (one-way or Welch’s ANOVA: F = 29.438, 7.488, 4.914, 13.480 and 16.396; all dfnum = 2; dfden = 12.633, 10.512, 11.222, 21 and 21; *p* < 0.001, =0.010, =0.029, <0.001 and <0.001). In the longitudinal comparison, the expression of *TNF-α* was significantly downregulated (t-test: t = 2.971; df = 14; *p* = 0.010), *IFN-γ* showed no significant change (t-test: t = −0.434; df = 14; *p* = 0.671), while *IL-8*, *TGF-β1*, and *IL-10* were significantly upregulated (t-test or Welch’s test: t = −2.425, −4.331 and −6.534; df = 7.524, 14 and 14; *p* = 0.043, 0.001, and <0.001) in the C9 group. In contrast, all five genes were significantly upregulated in both the M9 (t-test or Welch’s test: t = −6.254, −5.355, −7.288, −8.932 and −3.650; df = 7.654, 14, 8.212, 7.679 and 14; *p* < 0.001, <0.001, <0.001, <0.001 and =0.003) and N9 (t-test or Welch’s test: t = −6.258, −4.696, −5.634, −7.597 and −7.490; df = 7.441, 7.611, 7.040, 7.400 and 14; *p* < 0.001, =0.002, =0.001, <0.001 and <0.001) groups. The M9 group showed significantly higher upregulation of *TGF-β1* than the C9 group. The N9 group exhibited significantly greater upregulation of all five genes than the C9 group and the M9 group.

#### 3.5.2. Apoptosis-Related Genes

In the horizontal comparison, there were no significant differences in the expressions of *Caspase-3*, *Caspase-8*, *Caspase-9*, and Bcl-2-associated X protein (*Bax*) among the C3, M3, and N3 groups (Welch’s ANOVA: F = 2.994, 1.370, 1.087 and 2.434; all dfnum = 2; dfden = 12.451, 11.670, 12.444 and 11.904; *p* = 0.087, 0.292, 0.367 and 0.130). However, the expressions of the above four genes in the M9 (*p* = 0.048, 0.004, 0.043 and <0.001) and N9 (*p* = 0.001, <0.001, <0.001 and <0.001) group were significantly upregulated compared with those in the C9 group (one-way or Welch’s ANOVA: F = 9.131, 38.985, 32.362 and 44.600; all dfnum = 2; dfden = 21, 21, 21 and 11.237; *p* = 0.001, <0.001, <0.001 and <0.001). Compared with the M9 group, the expression levels of *Caspase-8* and *Caspase-9* were significantly upregulated in the N9 group (both *p* < 0.001). In the longitudinal comparison, the C9 group showed significant downregulation of the expressions of *Caspase-8*, *Caspase-9* and *Bax* (Welch’s test: t = 2.842, 3.506 and 5.864; df = 8.508, 8.401 and 9.805; *p* = 0.020, 0.007 and <0.001), while the expression of *Caspase-3* did not change significantly (t-test: t = 1.200; df = 14; *p* = 0.250). In the M9 group, the expressions of *Bax* was significantly upregulated (Welch’s test: t = 4.486; df = 9.744; *p* = 0.001). In the N9 group, the expressions of all the above four genes were significantly upregulated (t-test: t = −3.679, −3.002, −3.677 and −4.215; all df = 14; *p* = 0.002, 0.010, 0.002 and 0.001). For B-cell lymphoma 2 (*Bcl-2*), there were no significant differences in both horizontal (one-way ANOVA: F = 1.438 and 3.072; both dfnum = 2; both dfden = 21; *p* = 0.260 and 0.068) and longitudinal comparisons (t-test: t = 1.996, −0.099, and 0.365; all df = 14; *p* = 0.066, 0.922, and 0.720).

#### 3.5.3. Antioxidant-Related Genes

In the horizontal comparison, compared with the C3 group, the expressions of *GST-ω* and *Nrf-2* in the M3 group were significantly downregulated (*p* = 0.009 and 0.002). In the N3 group, the expressions of *CAT*, *GST-ω*, and *Nrf-2* were significantly downregulated (*p* = 0.004, 0.039 and 0.002) (one-way or Welch’s ANOVA: F = 57.442, 8.144 and 10.690; all dfnum = 2; dfden = 12.545, 12.880 and 21; *p* < 0.001, =0.005 and =0.001). Compared with the C9 group, the expressions of *SOD*, *CAT*, *GST-ω*, and *Nrf-2* were all significantly upregulated in both the M9 (*p* < 0.001, <0.001, <0.001 and =0.004) and N9 (*p* = 0.044, <0.001, <0.001 and <0.001) groups (one-way or Welch’s ANOVA: F = 49.049, 51.132, 106.377 and 12.737; all dfnum = 2; dfden = 21, 12.558, 11.845 and 21; all *p* < 0.001). Compared with the M9 group, the expressions of *SOD* and *CAT* in the N9 group were significantly downregulated (*p* < 0.001 and =0.017). In the longitudinal comparison, the expressions of *SOD*, *CAT*, and *Nrf-2* in the C9 group were significantly downregulated (t-test or Welch’s test: t = 4.787, 6.353 and 3.795; df = 14, 8.077 and 14; *p* < 0.001, <0.001 and =0.002). In the M9 group, the above three genes were significantly upregulated (t-test or Welch’s test: t = −5.022, −3.102, and −4.520; df = 14, 7.352 and 10.408; *p* < 0.001, =0.016 and =0.001). In the N9 group, except for *SOD*, the other two genes were also significantly upregulated (t-test: t = 5.281 and −4.301; both df = 14; *p* < 0.001 and =0.001).

### 3.6. Intestinal Microbiota

After filtering, a total of 11,940 ASVs (2,411,046 reads) were obtained, among which 6765 ASVs (147,254 reads) were identified as contaminant sequences (Table S3). The remaining 5175 ASVs (2,263,792 reads) were used for intestinal microbiota analysis (Table S4). The species accumulation boxplot, rarefaction curve, and abundance rank curve indicated that the sample size and sequencing depth were sufficient (Figure S2). The experimental and theoretical abundances of the positive controls at the phylum and genus levels are displayed in Figure S3A,C. The compositions of the top 10 phyla and genera with the highest abundances in the negative controls are shown in Figure S3B,D, and the absolute abundances and taxonomic information of their ASVs are listed in Table S5.

**Figure 5:**
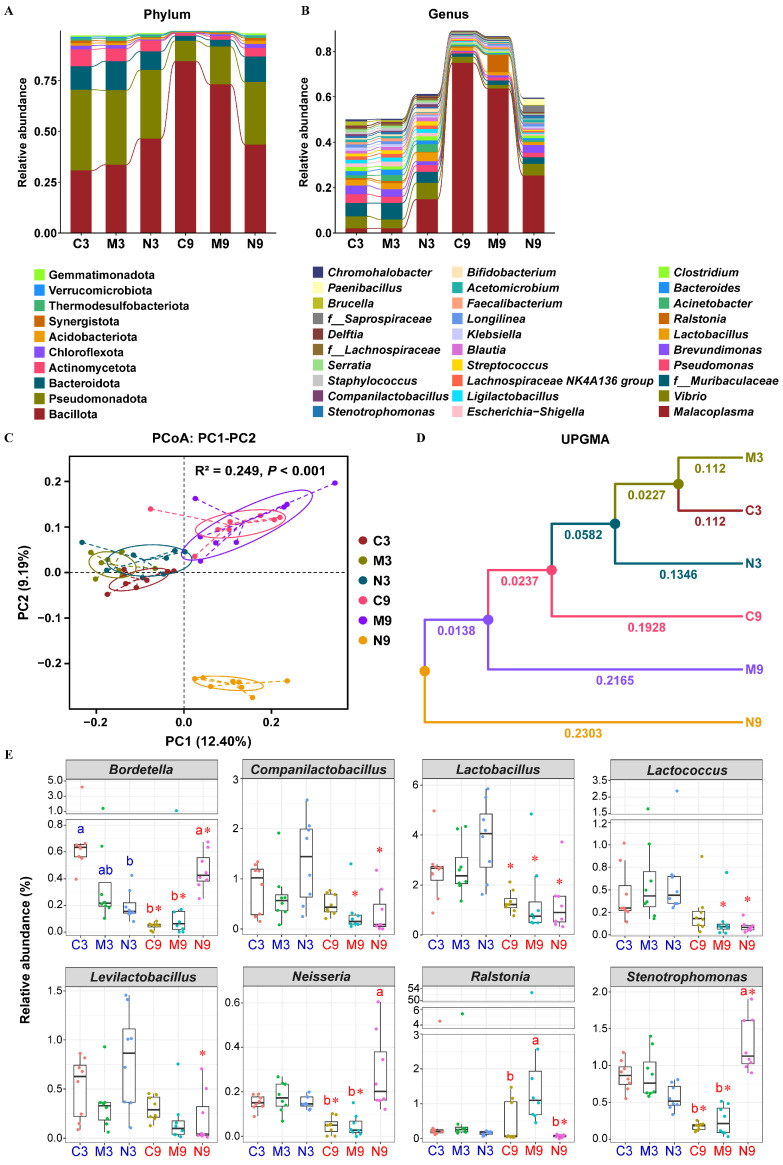
Effects of MPs and NPs on intestinal microbiota of turbot. C3, M3 and N3: control, MPs, and NPs groups in the 3rd week; C9, M9 and N9: control, MPs, and NPs groups in the 9th week. Top 10 most relative abundant at phylum level (**A**) and top 30 most relative abundant at genus level (**B**). Principal coordinate analysis (PCoA) plot in samples (**C**) and UPGMA clustering trees in groups (**D**) based on unweighted UniFrac distances. R^2^ > 0 indicates that the difference between groups is greater than that within groups; *p* < 0.001 indicates significant difference. Pathogenic bacteria and probiotics among differential features (**E**). ^a,b^ Values with different superscript letters (marked in the same color) indicate significant differences among treatments at the same time (*p* < 0.05). * Values marked with asterisks indicate significant differences over time within the same treatment (*p* < 0.05).

Regarding the intestinal microbiota of turbot, the top four phyla with the highest abundance across all groups were Bacillota (Average relative abundance (Ave) 51.96%), Pseudomonadota (Ave 28.26%), Bacteroidota (Ave 8.87%), and Actinomycetota (Ave 4.51%) (Figure 5A). The stacked abundance plot of the top 30 genera in terms of abundance across all groups (Figure 5B) revealed that the compositions of dominant genera in the C3 and M3 groups were highly similar, while the N3 group showed slight differences compared to these two groups. When compared with the C3, M3, and N3 groups respectively, the compositions of dominant genera in the C9, M9 and N9 groups all underwent significant changes. *Malacoplasma* became the absolute dominant genus in the C9 and M9 groups, with relative abundances as high as 75% and 64% respectively. Additionally, the composition of dominant genera in the N9 group was significantly different from that in the C9 and M9 groups. The PCoA analysis (Figure 5C), Adonis test results (Table S6) and UPGMA clustering tree (Figure 5D), all based on unweighted UniFrac distance, collectively indicated that there were differences in the community structure of intestinal microbiota among different groups. Specifically, the intestinal microbiota of all groups could be roughly divided into three clusters: the C3, M3, and N3 groups; the C9 and M9 groups; and the N9 group. The N9 group was clearly separated from the other two clusters. The MRPP results showed that in the comparisons of M3 vs N3 and C9 vs M9, A < 0 and *p* > 0.05, indicating that the intergroup difference was not significantly distinct from the intragroup difference, while in other comparisons, A > 0 and *p* < 0.05, indicating that the intergroup difference was greater than the intragroup difference.

The results of differential feature screening are presented in Table S7 and Figure 5E. The abundance of *Ralstonia* in the M9 group significantly increased compared to the C9 group. Compared with the C9 and M9 groups, the N9 group showed significantly increased abundances of pathogenic *Bordetella*, *Neisseria*, and *Stenotrophomonas*, with average relative abundances higher than those in the N3 group. Compared with the N3 group, the N9 group exhibited significantly decreased abundances of probiotic *Companilactobacillus*, *Levilactobacillus*, *Lactobacillus*, and *Lactococcus*, with average relative abundances lower than those in the C9 group; the average relative abundances of the latter two genera were also lower than those in the M3 group.

**Figure 6:**
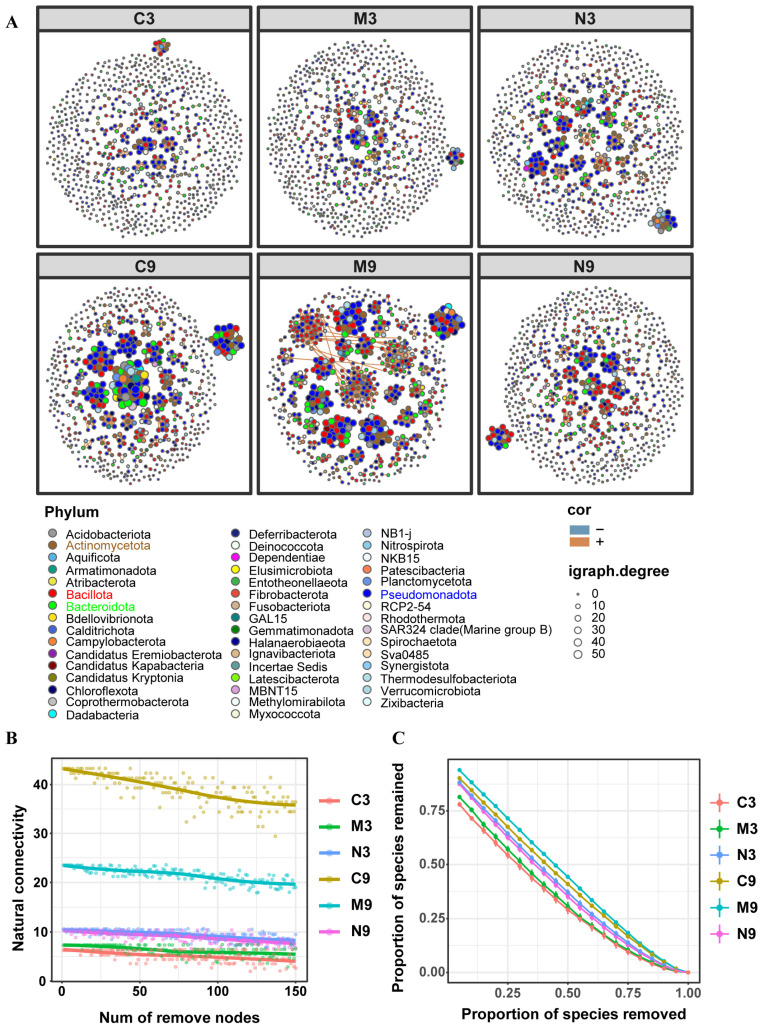
Effects of MPs and NPs on the microbial co-occurrence network. C3, M3 and N3: control, MPs, and NPs groups in the 3rd week; C9, M9 and N9: control, MPs, and NPs groups in the 9th week. Microbial co-occurrence network diagram (**A**). Natural connectivity of the network (**B**). Robustness of the network (**C**).

A total of 868 genus-level features were selected to construct microbial co-occurrence networks (Figure 6A). Differences between the E-R network and the co-occurrence networks indicated that the networks constructed for all groups were valid (Figure S4). Although most features in all groups showed positive correlations, there were differences in the properties and structures of the co-occurrence networks among different groups. Results of network parameters (Table 2) showed that in horizontal comparisons: For links, nodes, average degree, average path length, diameter, and relative modularity, the N3 group had higher values than the C3 and M3 groups; the M3 group had lower values than the C3 group only in the last two parameters. For links, nodes, average degree, and relative modularity, the N9 group had lower values than the C9 and M9 groups; the M9 group had lower values than the C9 group only in average degree. For average path length and diameter, the M9 group had the highest values, followed by the N9 group, and the C9 group had the lowest. Without considering negative links, longitudinal comparisons showed that in the C9 group, all parameters increased except average path length and diameter. All parameters increased in the M9 group, while all parameters decreased in the N9 group.

Although there were certain fluctuations in the natural connectivity of the networks in all groups during the node removal process, the overall trend was decreasing (Figure 6B). Overall, among the groups in the 3rd week, the natural connectivity of the N3 group was the highest, followed by the M3 group, and the C3 group was the lowest. Among the groups in the 9th week, the C9 group had the highest natural connectivity, followed by the M9 group, and the N9 group was the lowest. For longitudinal comparison, the natural connectivity of both the C9 and M9 groups increased significantly, while that of the N9 group decreased slightly. Regarding robustness (Figure 6C), after randomly deleting 50% of the nodes, among the groups in the 3rd week, the N3 group showed the highest robustness, followed by the M3 group, with the C3 group being the lowest. Among the groups in the 9th week, the M9 group had the highest robustness, followed by the C9 group, and the N9 group was the lowest. In longitudinal comparison, the robustness of both the C9 and M9 groups increased significantly, while the N9 group also decreased slightly.

## 4. Discussion

Currently, microplastics are widespread in aquaculture environments, but there is no unified benchmark concentration, and the concentrations of microplastics in water and sediments vary significantly across different aquaculture regions [10,11,12]. Nevertheless, it should be acknowledged that the 2% addition level employed in this study is considerably higher than the common pollution levels in actual environments and commercial feeds. Therefore, this study focuses on evaluating the toxic effects of microplastics on the growth and health of turbot, rather than conducting actual environmental risk assessments.

### 4.1. Growth Performance

Several studies have shown that both short-term and long-term exposure to MPs or NPs can inhibit the growth of aquatic animals [18,66,67]. However, other studies have found that the growth of *Lates calcarifer* [68] and marine medaka (*Oryzias melastigma*) [27] exhibits a certain tolerance to microplastics. Under the experimental conditions of the present study, continuous ingestion of feeds containing MPs or NPs for 3 and 9 weeks did not significantly affect the growth performance of turbot. This suggests that, compared with particle size and exposure duration, the growth tolerance of aquatic animals to microplastics may be more closely related to animal species, microplastic intake, and aquaculture environment [22,69].

### 4.2. Intestinal Health

Studies on gilthead seabream (*Sparus aurata*) have confirmed that irregularly edged, fragmented microplastics can induce intestinal tissue hemorrhage, epithelial cell detachment, and necrosis [16]. In the present study, however, MPs and NPs did not significantly affect the morphological structure of the turbot intestine. This is presumably because the microplastics used in the experiment were approximately spherical with smooth surface, resulting in minimal mechanical damage to the intestine. Notably, short-term exposure to MPs or NPs exacerbated intestinal inflammation in turbot, with NPs exerting a more significant effect. However, when the exposure duration was extended to 9 weeks, intestinal inflammation was significantly alleviated, indicating that the turbot intestine has a certain tolerance to the adverse stimulation of MPs and NPs.

MUC-2 is the primary mucin in the intestinal mucus layer [70]. Previous studies have shown that the expression of intestinal *MUC-2* in common carp is significantly upregulated after 2 weeks of MPs exposure [71]. In the present study, the expression of intestinal *MUC-2* in turbot was also significantly upregulated following 3 weeks of MPs exposure, whereas NPs did not induce this effect. This change is presumably a short-term adaptive response of the organism to MPs, which can resist their adverse effects on the intestine. Additionally, the particle size of microplastics is closely related to the induction of this adaptive response. A study on marine medaka found that MPs have a stronger effect on increasing intestinal mucus quantity than NPs [27], which is consistent with the results of the present study. Intestinal tight junctions are protein complexes that regulate the paracellular permeability of intestinal epithelial cells, with Claudin-3, Claudin-4, Tricellulin, and ZO-1 being their key constituent proteins [72]. Studies have shown that 21 days after gilthead seabream ingested MPs, the expression of multiple tight junction protein genes in the hindgut was significantly downregulated [73]. In the present study, MPs and NPs caused significant downregulation of intestinal *Claudin-3* (at 9th week) and *Claudin-4* (at 3rd week and 9th week) expression in turbot, while the expression of *Tricellulin* and *ZO-1* was not significantly affected. This indicates that although MPs and NPs exert a certain persistent damaging effect on the intestinal tight junctions of turbot, they do not cause the disintegration of their overall structure.

The accumulation of reactive oxygen species (ROS) is a primary cause of oxidative stress. Antioxidant enzymes (e.g., SOD, CAT, GPX, GST-ω) are key substances for scavenging ROS and repairing oxidative damage, while Nrf-2 can initiate the expression of antioxidant enzyme genes [74,75]. The gene expression levels of these substances are important indicators of the body’s antioxidant capacity. It is widely acknowledged that microplastics can induce oxidative stress in aquatic animals [76], however, their effects on antioxidant enzymes are highly complex [22]. Comprehensive findings from several studies have revealed that microplastics exert dual effects on the body’s antioxidant capacity, namely adaptive enhancement and inhibition [17,77,78]. In the present study, the intestinal antioxidant capacity of turbot decreased after 3 weeks of MPs and NPs exposure, but recovered to the level of the concurrent control group after 9 weeks of exposure. This indicates that the turbot intestine exhibits adaptable and recoverable antioxidant capacity under microplastic exposure.

### 4.3. Hepatic Health

Previous studies have confirmed that microplastics can penetrate the intestinal barrier of fish [79,80]. In the present study, the damaging effects of microplastics on the liver of turbot exhibited obvious size dependence and time-dependent cumulative effects: short-term exposure did not induce significant abnormalities in liver tissue, whereas long-term exposure caused marked hepatocyte damage, with NPs exerting a significantly stronger damaging effect than MPs. This discrepancy suggests that microplastic-induced liver damage requires a certain period of time, and NPs with smaller particle sizes may be more likely to penetrate the intestinal barrier, translocate to the liver, and thereby induce damage. Unfortunately, due to the characteristics of experimental materials and limitations of existing technical conditions, the present study did not detect microplastics in the liver tissues of turbot. While a variety of analytical approaches have been established for identifying and quantifying microplastics in biological tissues [81], the detection of nanoplastics remains fraught with substantial difficulties and technical limitations [82,83]. Additionally, differences in the uptake mechanisms of MPs and NPs by hepatocytes may also contribute to the more severe liver damage induced by NPs [84].

Pro-inflammatory cytokines (e.g., TNF-α, IL-8, IFN-γ) can initiate and enhance inflammation, while anti-inflammatory cytokines (e.g., TGF-β1, IL-10) balance inflammatory intensity and promote its resolution [85]. Microplastics also had significant size-dependent and time-dependent effects on the expression of inflammatory cytokine genes in the turbot liver. Specifically, no significant effects were observed after 3 weeks of exposure, while the expression of inflammatory cytokine genes were upregulated after 9 weeks of exposure, with NPs exerting a stronger effect than MPs. The results indicate that long-term intake of MPs and NPs can strongly activate hepatic inflammatory responses, thereby impairing liver health. Among them, NPs with smaller particle sizes deserve special attention.

Cell apoptosis is primarily executed through the exogenous death receptor pathway and the endogenous mitochondrial pathway. Caspase-8 initiates the exogenous pathway, while Caspase-9 triggers the endogenous pathway, both can activate the executioner molecule Caspase-3 to induce cell apoptosis. In the endogenous pathway, the anti-apoptotic protein Bcl-2 and the pro-apoptotic protein Bax collectively regulate the initiation of this pathway [86]. Studies on snakehead have demonstrated that 28-day exposure to MPs can induce hepatocyte apoptosis [17]. In the present study, the effects of microplastics on the expression of hepatic pro-apoptotic genes also exhibited obvious size dependence and time-dependent cumulative effects: 3-week exposure did not induce hepatic cell apoptosis in turbot, but after 9-week exposure, both the endogenous and exogenous apoptotic pathways in the liver were significantly activated, with NPs exerting a stronger effect than MPs.

The effects of microplastics on the expression of liver antioxidant-related genes in turbot also exhibit obvious size dependence and time-dependent cumulative effects. After 3 weeks of MPs or NPs exposure, the antioxidant capacity of the liver, similar to that of the intestine, decreased. However, after 9 weeks of exposure, the antioxidant defense response in the liver was significantly activated, with the effect in the NPs group being weaker than that in the MPs group. These results suggest that long-term microplastic exposure enhances oxidative stress-related responses in the liver of turbot. Possibly due to more severe liver damage (including disrupted cellular structures, inflammation and apoptosis) in the NPs group, the intensity of antioxidant defense was weaker than that in the MPs group. In addition, it should be stated that this study did not measure biochemical markers related to liver function and oxidative damage, which is a limitation of the present study.

### 4.4. Intestinal Microbiota

Several studies have found that microplastics exert negative effects on the intestinal microbiota of aquatic animals, manifested as altered diversity, increased abundance of pathogenic bacteria, and decreased abundance of probiotics [20,68,87]. However, research on the impacts of microplastics on the intestinal microbiota of aquatic animals under different particle size and exposure duration conditions remains limited. A study on zebrafish has shown that NPs are more likely to induce intestinal dysbiosis compared to MPs [26]. In the present study, both in the control group and the MPs and NPs exposure groups, the composition and structure of the intestinal microbiota of turbot changed significantly over time, indicating that time is the dominant factor leading to changes in the intestinal microbiota of turbot. Therefore, it is necessary to conduct comparisons among multiple time points when evaluating the effects of microplastics on the intestinal microbiota of aquatic animals.

Short-term exposure to MPs significantly altered the microbial community structure. It also increased the complexity and stability of the microbial co-occurrence network, as indicated by higher values of links, average degree, natural connectivity, and robustness. Long-term exposure to MPs did not significantly change the microbial community structure, but significantly increased the abundance of *Ralstonia*, a pathogenic genus containing species capable of causing infection and inflammation in various tissues [88]. Long-term exposure to MPs resulted in more complex changes in the microbial co-occurrence network: although links, relative modularity, and robustness were higher, the overall network structure became looser and microbial interactions were weakened, as reflected by lower average degree and natural connectivity, together with higher diameter and average path length [89].

However, both short-term and long-term exposure to NPs significantly altered the microbial community structure of turbot. Furthermore, long-term exposure to NPs resulted in significant dysbiosis of the intestinal microbiota in turbot. Specifically, NPs may have an enriching effect on *Bordetella*, *Neisseria*, and *Stenotrophomonas*. Although no studies have yet found that these three genera are pathogenic to fish, they are indeed pathogenic bacteria in humans and other mammals [90,91,92]. Therefore, their enrichment by NPs may pose a potential risk to human health. NPs may exert inhibitory effects on *Companilactobacillus*, *Levilactobacillus*, *Lactobacillus*, and *Lactococcus*. All these strains belong to lactic acid bacteria, a type of probiotics [93,94]. Regarding the microbial co-occurrence network, although short-term exposure to NPs increased network complexity and stability, as indicated by higher values of links, average degree, relative modularity, natural connectivity, and robustness, long-term exposure led to a significant reduction in its complexity and stability, characterized by lower values of the aforementioned five indices [89].

## 5. Conclusions

In conclusion, particle size and exposure duration are important factors regulating the impacts of PVC MPs and NPs on the intestinal and hepatic health of turbot. Although the growth performance and intestinal tissues of turbot exhibit a certain tolerance and adaptability to MPs and NPs, long-term exposure leads to significant liver damage, characterized by disrupted cellular structures, increased levels of inflammation and apoptosis, accompanied by activated antioxidant defense responses, with NPs exerting a stronger damaging effect. Compared with MPs, long-term exposure to NPs can induce obvious intestinal microbiota dysbiosis. In the future, particular attention should be paid to the long-term exposure risks of PVC NPs to aquatic animals.

## Figures and Tables

**Table 1:** The results of growth performance.

Indicator	C3	M3	N3	C9	M9	N9
IBW (g)	5.895 ± 0.009	5.898 ± 0.010	5.900 ± 0.006	5.895 ± 0.009	5.898 ± 0.010	5.900 ± 0.006
FBW (g)	13.05 ± 0.39	13.64 ± 0.39	13.14 ± 0.45	41.73 ± 1.70	44.51 ± 1.17	43.93 ± 1.35
WGR (%)	121 ± 7	131 ± 7	123 ± 8	608 ± 30	655 ± 21	644 ± 23
SGR (%/day)	3.78 ± 0.15	3.99 ± 0.14	3.80 ± 0.16	3.10 ± 0.07	3.21 ± 0.04	3.18 ± 0.05
FE	1.53 ± 0.10	1.49 ± 0.08	1.47 ± 0.04	1.42 ± 0.06	1.60 ± 0.20	1.52 ± 0.09
FI (%/day)	2.36 ± 0.08	2.55 ± 0.09	2.45 ± 0.05	1.69 ± 0.05	1.58 ± 0.15	1.61 ± 0.08
SR (%)	99.4 ± 0.6	99.4 ± 0.6	98.8 ± 0.7	99.3 ± 0.7	98.6 ± 0.8	98.6 ± 0.8

C3, M3 and N3: control, MPs, and NPs groups in the 3rd week; C9, M9 and N9: control, MPs, and NPs groups in the 9th week. Results are shown as means ± SE, *n* = 4. IBW: initial body weight; FBW: final body weight; WGR: weight gain rate; SGR: specific growth rate; FE: feed efficiency; FI: feed intake; SR: survival rate—the SR of each group at the 9th week was calculated after excluding individuals removed by dissection sampling at the 3rd week.

**Table 2:** Microbial co-occurrence networks parameters.

Parameters	C3	M3	N3	C9	M9	N9
Links	403	494	1033	2937	3158	918
Positive links	376	473	1016	2926	3153	906
Negative links	27	21	17	11	5	12
Nodes	288	324	416	462	617	398
Average degree	2.80	3.05	4.97	12.71	10.24	4.61
Average path length	1.057	1.059	1.654	1.018	5.646	1.196
Diameter	3.89	2.92	11.65	3.88	19.07	6.82
Relative modularity	0.73	0.70	1.58	2.60	2.94	1.35

C3, M3 and N3: control, MPs, and NPs groups in the 3rd week; C9, M9 and N9: control, MPs, and NPs groups in the 9th week.

## Data Availability

The original contributions presented in this study are included in the article and Supplementary Material. Further inquiries can be directed to the corresponding author.

## Supplementary Materials

The following supporting information can be downloaded at: https://www.mdpi.com/article/10.3390/toxics14040321/s1, Figure S1: Scanning Electron Microscopy (SEM) image of MPs (A), scale bar = 200 µm. SEM image of NPs (B), scale bar = 8 µm. Histogram of particle size frequency distribution of MPs (C) and NPs (D); Figure S2: The species accumulation boxplot (A), rarefaction curve (B), and abundance rank curve (C); Figure S3: Microbial composition at phylum and genus levels in positive and negative control samples. Act. denotes the actual value, and Nom. denotes the nominal value; Figure S4: E-R network and co-occurrence networks; Table S1: The sequences and amplification efficiencies of qPCR primers; Table S2: Stability of reference genes; Table S3: The absolute abundance and taxonomic information of ASVs identified as contaminated; Table S4: The absolute abundance and taxonomic information of ASVs for intestinal microbiota analysis; Table S5: The absolute abundance and taxonomic information of ASVs in negative controls; Table S6: Adonis tests and MRPP of the microbial community structure of turbot; Table S7: Differential features information.
