# Supplementary material for: Size- and Time-Dependent Impacts of Polyvinyl Chloride Microplastics on Turbot (Scophthalmus maximus L.): Intestinal Tolerance, Hepatic Injury, and Intestinal Microbiota Dysbiosis
Source: Toxics. 2026 Apr 12;14(4):321. doi: 10.3390/toxics14040321 (PMC13120644; doi:10.3390/toxics14040321)
Supplement: Supplementary file 1 [file toxics-14-00321-s001.zip › Supplementary Figures.pdf]

# Supplementary Figures

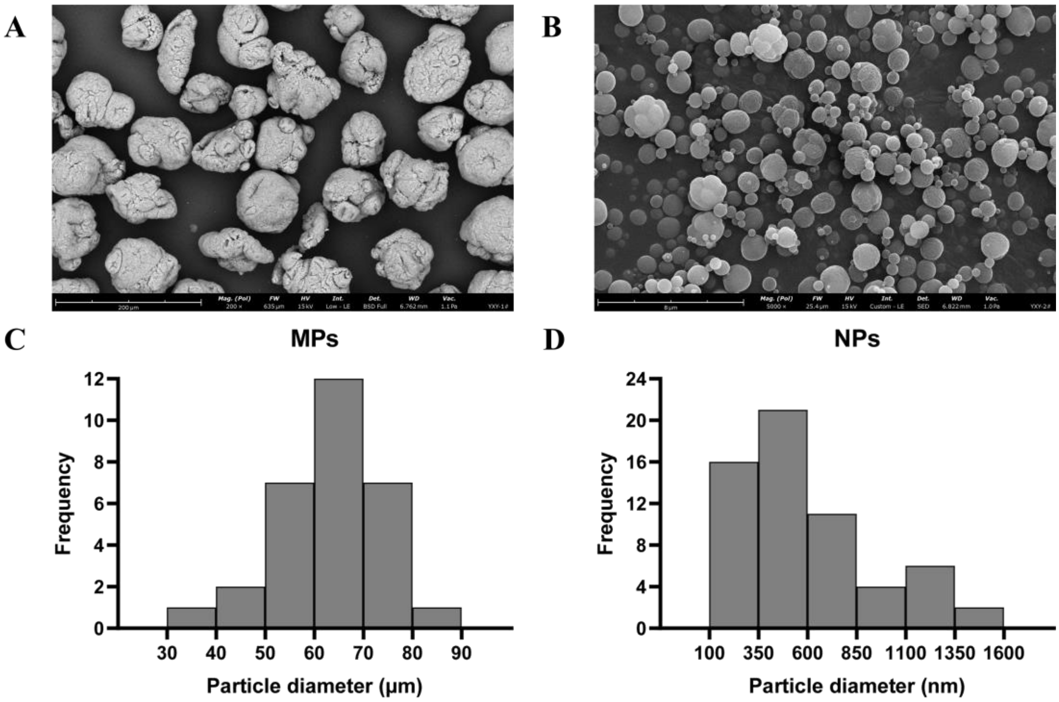


**Figure S1.** Scanning Electron Microscopy (SEM) image of MPs (A), scale bar = 200 μm. SEM image of NPs (B), scale bar = 8 μm. Histogram of particle size frequency distribution of MPs (C) and NPs (D).

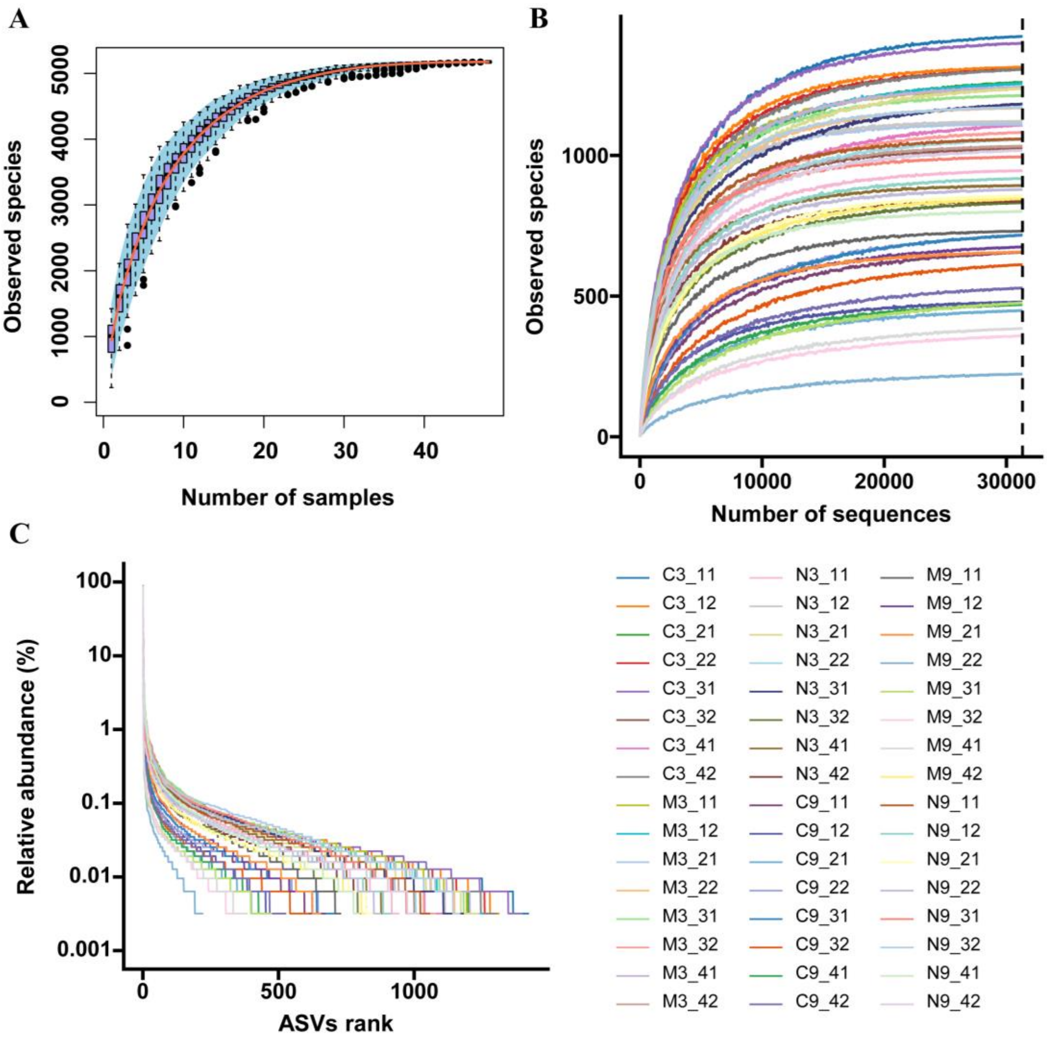


**Figure S2.** The species accumulation boxplot (A), rarefaction curve (B), and abundance rank curve (C).

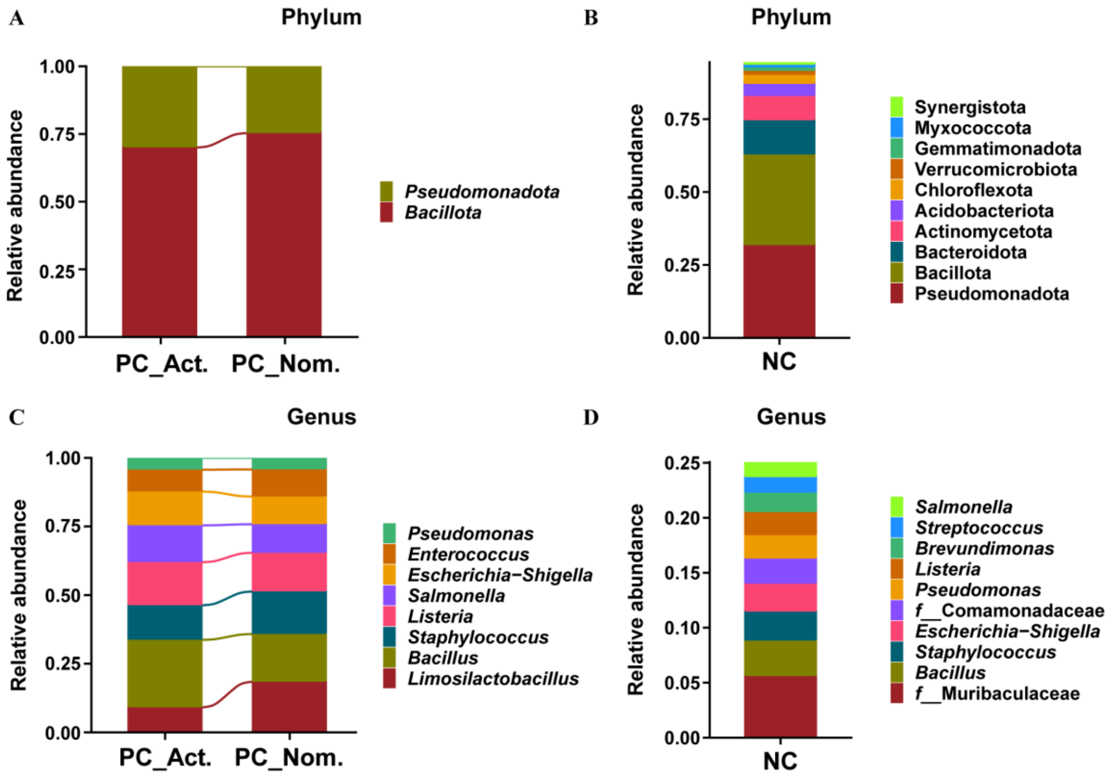


**Figure S3.** Microbial composition at phylum and genus levels in positive and negative control samples. Act. denotes the actual value, and Nom. denotes the nominal value.

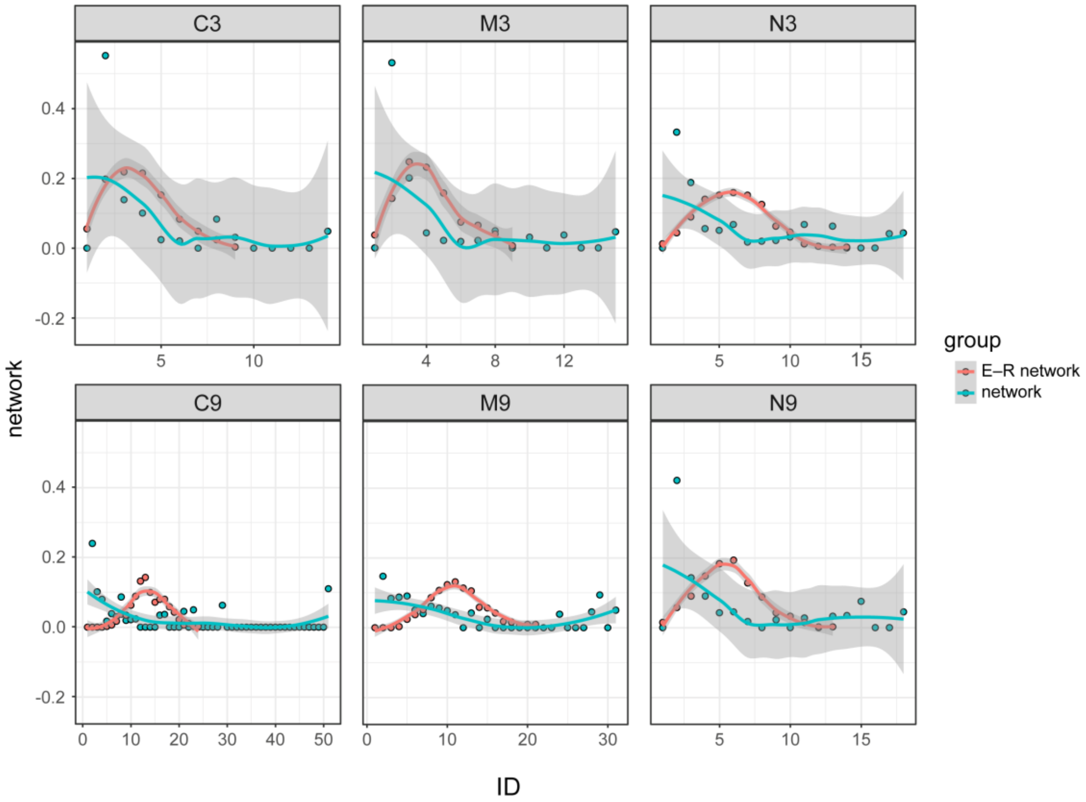


**Figure S4.** E-R network and co-occurrence networks.
